# Using Wearable Sensors to Study Musical Experience: A Systematic Review

**DOI:** 10.3390/s24175783

**Published:** 2024-09-05

**Authors:** Erica Volta, Nicola Di Stefano

**Affiliations:** Institute of Cognitive Sciences and Technologies, National Research Council, Via Gian Domenico Romagnosi 18/A, 00196 Roma, RM, Italy; erica.volta@itd.cnr.it

**Keywords:** audio-tactile mapping, gesture recognition, music interaction, music performance, wearable sensors, wearable technologies

## Abstract

Over the last few decades, a growing number of studies have used wearable technologies, such as inertial and pressure sensors, to investigate various domains of music experience, from performance to education. In this paper, we systematically review this body of literature using the PRISMA (Preferred Reporting Items for Systematic Reviews and Meta-Analyses) method. The initial search yielded a total of 359 records. After removing duplicates and screening for content, 23 records were deemed fully eligible for further analysis. Studies were grouped into four categories based on their main objective, namely performance-oriented systems, measuring physiological parameters, gesture recognition, and sensory mapping. The reviewed literature demonstrated the various ways in which wearable systems impact musical contexts, from the design of multi-sensory instruments to systems monitoring key learning parameters. Limitations also emerged, mostly related to the technology’s comfort and usability, and directions for future research in wearables and music are outlined.

## 1. Introduction

The rapid advancement of wearable technology has opened up new possibilities across various research fields [1,2], including music [3]. This systematic literature review explores how wearable technologies are used within current research in musical contexts, focusing especially on their applications, benefits, and potential challenges. By analyzing a diverse range of studies, this review seeks to provide a comprehensive understanding of how these technologies are being integrated into music performance, education, and therapy, among other areas.

The intersection of wearable technology and music has become a promising field of interest, driven by the increased economic accessibility of these technologies and the proliferation of high-quality commercial platforms [2,3,4]. These advancements have democratized wearable technologies, allowing even non-technical experts to incorporate them into their artistic or experimental settings. Furthermore, there is a growing emphasis on the ergonomics of wearable devices and their accessibility for individuals with sensory and motor impairments that further helps to improve the research on the field [5,6].

In recent years, the utilization of music as a stimulus within therapeutic and rehabilitative settings has garnered significant attention. A range of studies highlighted the promising role of music in engaging with special needs populations, including autistic children, and patients recovering from conditions like Parkinson’s disease or strokes. For example, recent advancements have seen the development of systems that use music and rhythmic stimuli to aid in the motor rehabilitation of stroke survivors by synchronizing patient movements with musical cues to improve motor outcomes [7]. Similarly, research has shown that auditory feedback can significantly enhance motor learning in children with cerebral palsy, leveraging music within a dance-based therapy paradigm to motivate and guide movement [8]. In contexts involving autism spectrum disorder (ASD), affective computing technologies utilize music to respond to and modulate the emotional states of children, tailoring therapeutic interactions based on real-time physiological data [9]. These systems often employ wearable technologies to monitor emotional or physiological responses, adjusting musical stimuli to either enhance therapeutic engagement or mitigate distress. This approach not only deepens our understanding of the interplay between sensory stimuli and therapeutic outcomes but also exemplifies the potential of integrating wearable tech with music therapy to offer personalized support. While the evidence underscores music’s efficacy as both a rehabilitative tool and a form of emotional feedback, our review focuses on a slightly different facet of wearable technology in music contexts. Specifically, we examine how these technologies are employed to enhance musical performance, interaction, and education, rather than their use as therapeutic stimuli in medical or rehabilitative settings. Consequently, studies primarily investigating music as a stimulus for therapeutic purposes are outside the scope of this review. This focus allows us to explore the technological advancements and their applications in enhancing musical engagement and pedagogy in depth, paving the way for future research that might bridge these distinct but potentially complementary areas of study.

Recent studies have explored wireless wearable sensing devices in interactive music, demonstrating how machine learning can enhance gesture recognition accuracy and create immersive experiences (e.g., refs. [3,10]). These advancements allow for real-time feedback and adaptability, crucial for enhancing technical precision and artistic expression in musical performances. Moreover, the use of multi-node wearable systems has been shown to significantly improve music performance by providing detailed feedback on musicians’ movements and techniques, facilitating training and skill enhancement [11]. Furthermore, the development of accessible digital musical instruments is another area of interest worth exploring (e.g., ref. [5]), as well as the design of innovative wearable instruments (e.g., the Serendiptichord, see [12,13]).

Studies have also delved into the physiological aspects of music performance, using wearable sensors to monitor stress responses and understand the physical demands placed on musicians. Research has shown how these devices can track physiological parameters such as heart rate and stress levels, providing insights into managing performance anxiety and improving overall well-being [14,15,16].

Gesture recognition remains a critical area of exploration, with wearable technologies capturing and classifying physical gestures in musical applications. Utilizing systems like EMG sensors and the Myo armband, these studies highlight how wearable technologies can facilitate new forms of musical interaction and expression [17,18]. The integration of wearable technologies in dance and music performances further emphasizes the potential for creating immersive and interactive environments [6].

Despite the growing interest and significant progress in this field, however, several challenges persist. Issues related to calibration mechanisms, user comfort, and long-term usability require further attention. These challenges, along with potential future research directions, will be discussed in Section 5, while in Section 6 we will try to advance some hypotheses of future directions on wearable research and development.

By synthesizing the findings from these diverse studies, this review aims to elucidate the current landscape of wearable technology in music, identify key trends and gaps, and propose directions for future research. The goal is to provide a study that can serve as a basis for researchers and practitioners interested in the intersections of technology and music, ultimately contributing to advancing this interdisciplinary and rapidly growing field.

## 2. PRISMA Review

Following the guidelines suggested in the PRISMA method [19], we systematically review the research on the use of wearable technologies in the music context from the first identified article published back in 1999 through June 2024. The document search was conducted through Scopus and Web of Science databases using the following string: “wearable technolog*” OR “wearable sensor*” OR “wearable*” OR “imu” OR “mimu” AND “music*”. The inclusion criteria were as follows: full text available, written in English, and empirical articles. The exclusion criteria were defined as well: books, commentaries, conference reports, editorials, articles in languages other than English, and gray literature (non-academic reports or documents, or any other material that did not pass the criteria for inclusion). The review was registered in INPLASY with the registration number INPLASY202470098 on 24 July 2024.

**Figure 1:**
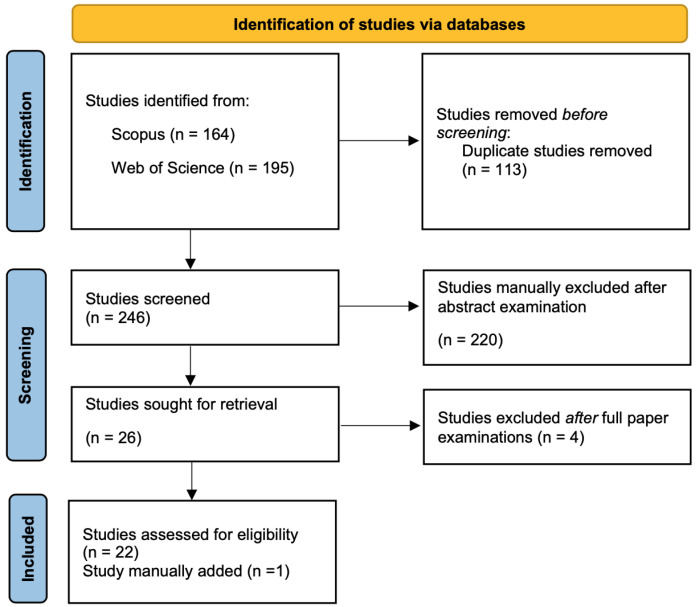
PRISMA diagram showing the databases searching process and the screening performed.

**Figure 2:**
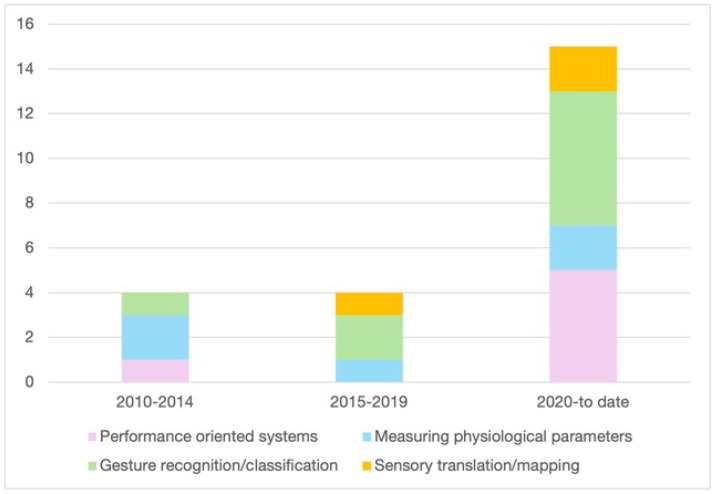
Publication trends since 2010 are divided into the four categories identified in this review.

The initial search yielded a total of 359 records. Of these, 118 records were immediately excluded as duplicates. We then screened the titles and abstracts of the remaining 241 articles. A total of 215 were manually excluded for using music solely as an experimental stimulus or feedback, focusing on the use of music for therapy (and not for performance), being theoretical articles, review or pre-print articles, missing fundamental information (i.e., authors), or not being related to the research question. The remaining 26 records were considered fully eligible as meriting further analysis. One additional paper was manually added because it was published after the database search, and four papers were excluded after a full examination as they considered the use of wearable technologies mainly for artistic purposes. This process resulted in 23 records entering the analysis (see Figure 1). Articles were categorized according to the following categories: performance-oriented systems (n = 6); measuring physiological parameters (n = 5); gesture recognition/classification (n = 9); sensory translation/mapping (n = 3) (see Figure 2 below). In Table 1, the 23 papers considered for the review are listed. The trend of publications demonstrates that the interest in this topic started about 15 years ago, with the number of papers published remarkably increasing since 2020.

**Figure 3:**
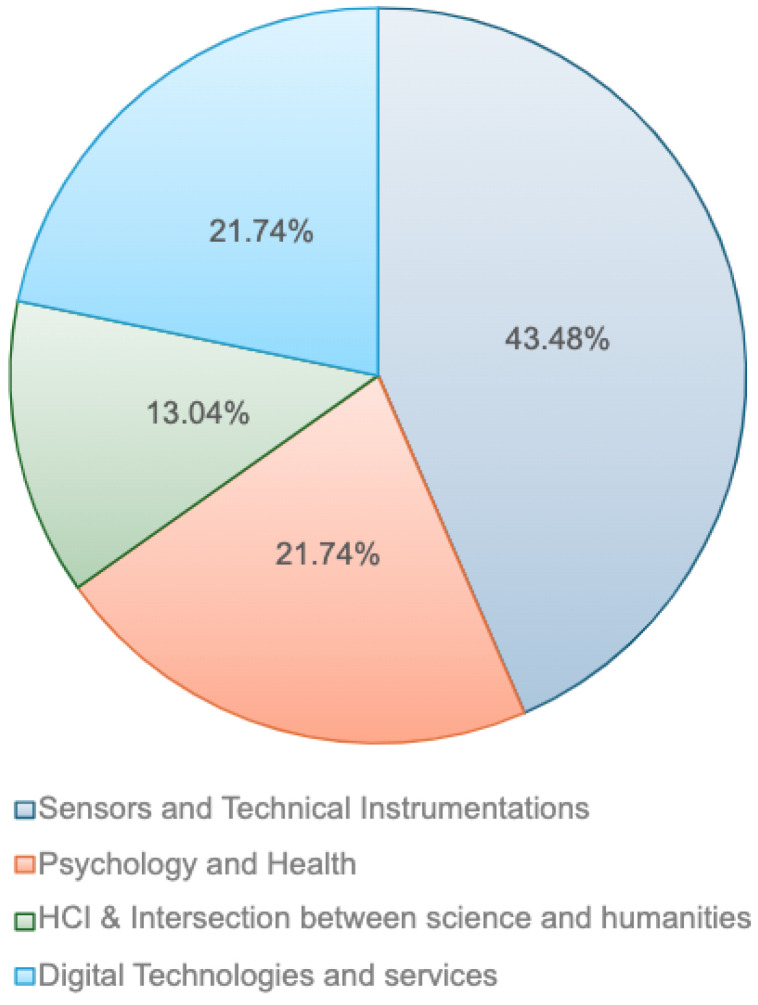
Pie chart showing the percentage of papers published within each of the four categories.

Regarding publication venues, as illustrated in Figure 3, approximately 43.5% of the papers considered in this study appeared in journals specialized in sensors and technical instrumentation (e.g., *IEEE Transactions on Instrumentation and Measurement, IEEE Sensors Journal, IEEE Transactions on Consumer Electronics, Sensors, Wearable Technologies*); about 22% were published in journals related to psychology and health (e.g., *Frontiers in Psychology, Medical Problems of Performing Artists, Music Perception*), and the same percentage in digital technologies and services journals (e.g., *Entropy, Personal and Ubiquitous Computing, Scientific Programming, Journal of Advances in Information Technology, Internet Technology Letters*). The remaining 13% were published in venues focused on human-computer interaction and more generally on the intersection of sciences and humanities (e.g., *Leonardo, IEEE Transactions on Human-Machine Systems, Multimodal Technologies and Interaction*). This distribution reflects the expectations, highlighting that 43% of the articles were published in technical venues specialized in sensors, while slightly more (57%) were published in journals focusing on the application and study of sensors-based technologies. This emphasizes the strategic importance of researching not only in the development of sensors but also in their application areas, underscoring the interdisciplinary nature and broad relevance of these technologies.

## 3. Results

This section presents a comprehensive analysis of the selected papers, divided into the four categories introduced above, namely “performance-oriented wearable systems”, “measuring physiological parameters”, “sensory translation/sensory mapping” and “gesture recognition/classification.” Each subparagraph delves into the contributions and insights offered by the studies within these categories, highlighting the advancements and potential of wearable systems in enhancing musical performance, gesture recognition in performance and education, interaction, and inclusivity.

**Figure 4:**
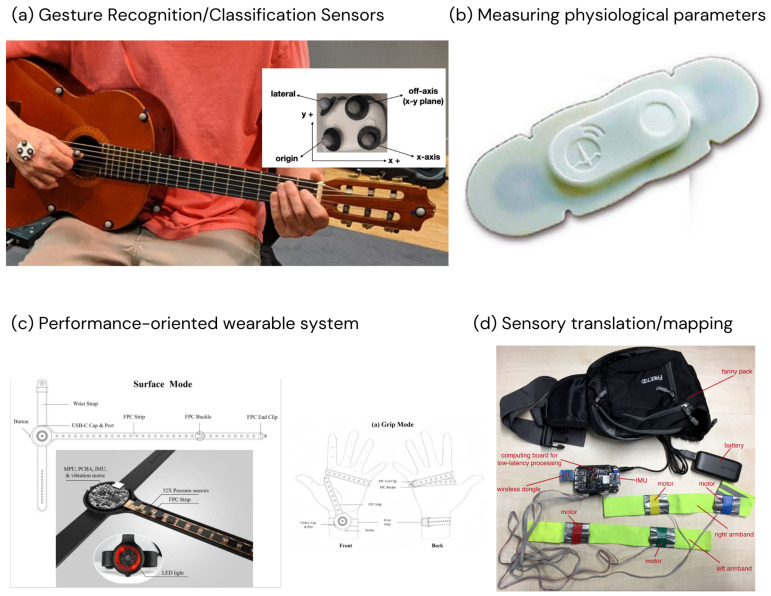
The image presents four sensors, one for each of the four categories considered in this review. In particular, for category (**a**) gesture recognition/classification, we have shown a sensor taken from [6], where the technical setup comprised an inertial sensor (MetaMotionR from MBIENTLAB, San Jose, CA, USA) and a high-end commercial Qualysis motion capture system (Qualisys AB, Göteborg, Sweden), typically used in the controlled environments of laboratories research. For category (**b**) measuring physiological parameters, we have shown a sensor taken from [14], where the sensor equipment comprised a VitalConnect HealthPatch® (VitalConnect, Campbell, CA, USA), an oblong adhesive biosensor patch containing two ECG electrodes, a thermistor, and an accelerometer. For category (**c**) performance-oriented wearable systems, we have shown the sensor used in [3], a wearable device prototype that contained 32 individual touch-sensitive pressure sensors, a nine-axis inertial-measurement-unit motion sensor, and various light-emitting diode and vibrational haptic-feedback components. Finally, for category (**d**) sensory translation/sensory mapping, we have shown the sensor used in [27], where the technical equipment comprised a prototype of musical haptic wearables for audiences (MHWA). From a hardware point of view, it is composed of a small fanny pack; two elastic armbands; a Bela board for low-latency audio processing; a Wi-Fi USB dongle; four vibration motors, two for each armband; and a lightweight power supply.

For illustrative purposes, Figure 4 shows some of the sensors used in the papers selected for the review. Specifically, the image presents one sensor for each category into which the papers have been grouped. The sources are cited in the caption of the figure.

### 3.1. Performance-Oriented Systems

The category “performance-oriented wearable systems” brings together a rich tapestry of research that highlights the transformative potential of wearable technologies in music performance, and leverages this technology to transform music performance through enhanced interaction, accessibility, and real-time feedback.

Wang [10] investigated the application of wireless wearable sensing devices in interactive music, with a strong emphasis on the role of machine learning. By employing multi-sensor fusion algorithms and data dimension reduction techniques, Wang’s study demonstrates how machine learning can effectively analyze the data from these devices to enhance the accuracy of gesture recognition and improve the overall interactive experience for both performers and audiences. This work underscores the importance of real-time data processing in creating immersive and responsive musical environments. Building on this technological foundation, Wexler and colleagues [3] explored the innovative applications of wearable technology across various musical contexts, focusing particularly on creating a new musical instrument that simplifies music learning and expression and makes them more intuitive. The authors demonstrated how wearable devices can be seamlessly integrated into different performance settings to enhance artistic expression and technical precision. Li [11] furthered this exploration by focusing on the enhancement of music performance through multi-node wearable sensors. Their research employs advanced data processing techniques such as generalized discriminant analysis (GDA) to reduce the high dimensionality of sensor data while retaining crucial performance traits. The result is a system that provides musicians with detailed feedback on their movements and techniques, facilitating significant improvements in performance skills.

Cavdir [5] took a unique approach by developing accessible digital musical instruments specifically designed for the deaf and hard-of-hearing community. This research highlights the inclusive potential of wearable technology, showcasing how these systems can enable individuals with hearing impairments to engage with music in new and meaningful ways. The participatory design process employed by the authors ensures that the resulting instruments are not only functional but also tailored to the needs and preferences of their users. Furthermore, Murray-Browne and colleagues [12] introduced the Serendiptichord, a wearable instrument designed for dance-driven interactive music systems. This device allows performers to generate sound through their movements, effectively merging the fields of dance and music. The Serendiptichord exemplifies the seamless integration of physicality and musical expression, providing a compelling example of how wearable technology can expand the boundaries of traditional performance art.

Finally, Pras [13] explored the integration of digital wearables in improvisational drumming. Their research focuses on how these technologies can augment traditional drumming techniques, allowing performers to experiment with new forms of creative expression. By incorporating wearables into the drumming process, the authors aimed to demonstrate how technology can enhance the spontaneity and dynamism of improvisational performance. Together, these studies illustrate the vast potential and the growing interest in the field of performance-oriented wearable systems to revolutionize music performance. From the application of machine learning for real-time data processing to the creation of inclusive instruments and the fusion of physical and musical expression, wearable technologies are paving the way for new and exciting developments in musical performance.

### 3.2. Measuring Physiological Parameters

This category encompasses a variety of research efforts that aim at understanding the relationship between music performance and physiological responses, particularly focusing on stress, emotional engagement, and the overall well-being of musicians.

Van Fenema and colleagues [14] comprehensively examined performance-induced stress among music students by employing wearable biosensor patches to measure key physiological parameters such as heart rate, respiratory rate, and skin temperature. Their findings reveal a marked increase in these parameters during live performances compared to resting states, underscoring the heightened stress levels musicians experience on stage. Their study also highlights a disconnection between physiological data and self-reported stress levels via psychometric questionnaires, suggesting the complexity of accurately gauging subjective stress. Building on this understanding, Sebastiani [15] delved into the synchronization between music dynamics and heart rhythm, exploring how a musician’s emotional involvement modulates this relationship. By analyzing heart rate variability (HRV) in a pianist playing various classical and jazz pieces, they demonstrate that physiological responses are significantly influenced not only by the technical demands of the music but also by the musician’s emotional engagement. This study underscores the nuanced interplay between physiological states and musical expression, revealing how deeply interconnected they are.

Kim and colleagues [16] extended the exploration of stress in musicians by focusing on using wearable sensor systems to monitor stress responses. Their study employs advanced classification techniques to analyze physiological signals such as heart rate and body motion to better understand and manage performance anxiety. The real-time feedback provided by these wearable systems offers practical solutions for musicians to effectively manage stress, ultimately aiming to enhance their performance quality and overall well-being.

Marrin Nakra and colleagues [22] introduced an innovative approach with the Serendiptichord, the device presented above. While primarily the Serendiptichord is a tool for creative performance, it also offers valuable insights into the physiological aspects of performance. By enabling performers to generate sound through their movements, this device allows researchers to investigate physical exertion and creative expression simultaneously, providing a unique perspective on the physiological demands of artistic performance. Moreover, Kusserow and colleagues [21] focused on the practical application of wearable sensors to measure stress levels in musicians during public performances. Their research includes monitoring heart rate and body motion to assess the impact of stage fright and other stressors on performance quality. By providing real-time data and feedback, these sensors help musicians develop strategies to cope with performance anxiety, highlighting the potential of wearable technology to support mental and physical health in high-pressure situations.

Collectively, these studies offer a detailed picture of the advancements in measuring physiological parameters in the context of musical performance. They reveal the significant impact of wearable technology in the field since they provide objective data that can inform strategies for managing stress and enhancing performance. These insights not only contribute to our understanding of the physical and emotional demands placed on musicians, but also pave the way for innovative approaches to support their health and artistic output. Through the integration of biosensors, machine learning, and interactive technologies, researchers are developing comprehensive tools that offer real-time analysis and feedback, helping musicians to thrive in their craft with better stress management and enhanced performance capabilities.

### 3.3. Gesture Recognition/Classification

#### 3.3.1. Performance

The category “gesture recognition/classification” and particularly the subsection related to “performance” delves into the use of wearable technologies to capture, analyze, and interpret physical gestures in musical and performance contexts. These studies demonstrate significant advancements in integrating sensors and machine learning to enhance interactive experiences and performance quality.

Maragliulo [17] presented a dual-channel wearable EMG system designed for precise foot gesture recognition. This system utilizes electromyography (EMG) sensors to detect muscle activity, allowing for the accurate identification of various foot gestures. By employing support vector machine (SVM) classifiers, the study achieved impressive accuracy in gesture classification, highlighting the potential of wearable EMG systems in enhancing human-computer interaction and musical performances. The research highlighted the importance of capturing detailed physiological data to improve the reliability and usability of gesture-based interfaces, opening new possibilities for interactive music applications. Rhodes et al. [18] explored the Myo armband, a wearable device equipped with both EMG and inertial measurement unit (IMU) sensors, for gesture recognition in music applications. Their study evaluated multiple machine-learning models to identify the most effective methods for classifying musical gestures. The combination of EMG and IMU data provided a comprehensive analysis of hand and arm movements, offering a robust framework for developing interactive music systems. The findings underscored the critical role of machine learning in processing complex sensor data to achieve reliable and accurate gesture recognition, enhancing the interactive capabilities of musical performances.

Li et al. [29] focused on integrating multi-node wearable sensor data to enhance music performance skills. Utilizing advanced data processing techniques such as generalized discriminant analysis (GDA), the study effectively reduced the high dimensionality of sensor data while preserving essential performance traits. This approach enabled the system to provide detailed, real-time feedback on musicians’ movements, facilitating significant improvements in both technical and expressive aspects of performance. The research emphasized the transformative potential of wearable sensors in offering actionable insights that enhance the quality of musical performance.

Finally, Freire et al. [6] investigated the integration of wearable technologies in dance and music performances, highlighting how these sensors can capture intricate dancer movements and translate them into interactive musical elements. By integrating wearable technologies, the study created a dynamic and immersive performance environment where dance movements directly influence the musical output. This research exemplified the seamless fusion of dance and music through technology, showcasing the innovative possibilities of wearable systems in performance art.

These studies illustrated the potential of wearable technologies in gesture recognition and classification within the fields of music and performance. Leveraging advanced sensors and machine learning algorithms, these research efforts enhanced the interactive capabilities and performance quality of musicians and dancers. The integration of physiological data and movement analysis not only improved the accuracy of gesture recognition but also opened new avenues for creative expression and interactive experiences. Through these advancements, wearable technology continued to push the boundaries of traditional performance art, enabling more immersive, responsive, and innovative artistic practices. This body of work highlighted the crucial intersection of technology and art, demonstrating how wearable systems can revolutionize the way we understand and engage with music and performance.

#### 3.3.2. Education

Van der Linden [20] presented ‘MusicJacket’, a wearable system designed for novice violin players. Leveraging an inertial motion capture system, the MusicJacket allows the tracking in real-time of postures and bowing parameters. Learners receive vibrotactile feedback about their bowing and posture through vibration motors positioned on their arms and torso. Comparing a sample of novices who used MusicJacket with a control group, the authors demonstrated that vibrotactile feedback effectively improves novices’ straight bowing technique. Notably, half of these subjects continued to show improved bowing technique even when they no longer received vibrotactile feedback. In contrast, none of the control subjects, who received the same number of training sessions using conventional teaching techniques, showed a comparable improvement.

Two studies targeted the automated classification of bowing gestures in violinists. Dalmazzo [24] presented a machine-learning approach to automatic violin bow gesture classification based on hierarchical hidden Markov models (HHMM) and motion data. Motion and audio data of a professional violinist were gathered to classify several bow techniques (i.e., détaché, martelé, spiccato, ricochet, sautillé, staccato, and bariolage). Motion data were recorded using a commercial Myo placed on the right forearm. Motion data were synchronized with audio recordings. After extracting features from both the motion and audio data, they trained an HHMM to identify the different bowing techniques automatically. The model can determine the studied bowing techniques with over 94/100 accuracy. The results make feasible the application of this work in a learning scenario, where violin students can benefit from the real-time feedback provided by the system.

In a similar study, Muhammed et al. [28] used one convolutional neural network (CNN) and two long short-term memory (LSTM) models to classify bowing gestures into one of the five bowing classes: detaché, legato, martelé, spiccato, and staccato. The dataset included audio samples performed by eight violinists and the motion data of their forearms measured using a Myo sensor device to acquire eight channels of electromyogram (EMG) data and 13 channels of inertial measurement unit (IMU) data. The audio and motion features are passed into an ensemble of deep-learning models to make the final prediction using weighted voting. The proposed ensemble classifier was able to deliver optimal results with an overall accuracy of 99.5/100.

The two studies by Provenzale and colleagues focus on monitoring bowing gestures in violin learners. In the first study [25], a system based on a magneto-inertial measurement unit (MIMU) and an optical sensor interface was designed and tested with beginner violinists for real-time monitoring of fundamental bowing parameters. Two MIMUs and a sound recorder were used to estimate bow orientation and acquire sound data, while an optical motion capture system served as the gold standard for comparison. Four optical sensors positioned on the bow stick measured the stick–hair distance. During a pilot test, a musician performed strokes using different sections of the bow at varying paces. Data analysis on nine violin beginners indicated that the interface provides reliable information on the bowing technique, which could enhance the learning performance of violin beginners.

In the second study [30], the researchers addressed one of the limitations of traditional teaching methods, namely the need for constant teacher supervision and the interpretation of non-real-time feedback provided after the performance. They introduced a novel interface, the visual interface for bowing evaluation (VIBE), to facilitate student progression throughout the learning process, even without direct teacher intervention. This interface monitors two key parameters of bowing movements: the angle between the bow and the string and the bow tilt, providing real-time visual feedback on correct bow movements. Results from 24 beginners (12 exposed to visual feedback, 12 in a control group) showed a positive effect of real-time visual feedback on bow control improvement. Additionally, the subjects exposed to visual feedback found it useful for correcting their movements and being clear in terms of data presentation. Importantly, the subjects did not feel the presence of a violin teacher was essential for interpreting the feedback.

In conclusion, various studies have demonstrated the effectiveness of innovative technologies in improving bowing techniques for violin learners. The MusicJacket and VIBE systems highlight the benefits of real-time vibrotactile and visual feedback, significantly enhancing novice violinists’ performance compared to conventional methods. Machine learning approaches have shown high accuracy in classifying bowing gestures, proving useful for automated training scenarios. The integration of motion and audio data facilitates precise feedback, aiding students in self-correction and skill improvement. These advancements suggest a promising future for technology-assisted music education, reducing the reliance on constant teacher supervision.

### 3.4. Sensory Translation/Mapping

In recent years, the possibility of conveying the information carried by one sensory input through another or, at the very least, of providing a sensory impression of one modality employing a sensation that is normally associated with the stimulation of another modality, has long attracted the interest of several scholars and artists. Spence and Di Stefano [31] refer to this topic in terms of ’sensory translation’ noticing that, beyond its application to artistic performance or marketing/design contexts, the topic is worth investigation in itself for the number of intriguing (not to mention challenging) theoretical and practical issues it raises. Thus, despite being numerically less important than the other categories, the category of studies on sensory translation or mapping is probably the most relevant conceptually. While not presenting themselves as studies on the topic of sensory translation, all of the studies falling in this category present a clear aspect in which the mapping between the senses is fundamental for obtaining their scope.

In the study by Turchet [23], researchers adopted a user-centered design methodology to develop a novel class of IoT devices that should facilitate communication among performers. Based on workshops conducted with performers in electronic music practice, they developed three chest-, foot-, and arm-worn haptic wearables for different forms of interactions, namely, co-performer, performer–conductor, and performer–sound-engineer. In the latter case, for example, performers exchanged information using tactile cues via a haptic belt and delivered information to the sound engineer, who captured the information through the foot-worn system. These foot-worn wearables include two motors placed on opposite sides of the ankle, with each motor being associated with a different musician according to their position to the location of the sound engineer. Using the buttons, the performers can communicate the following information to the sound engineer. The results provide evidence that musical haptic wearables can be an effective medium of communication in the context of electronic music performances. In a subsequent study, Turchet [27] shifted their attention towards the audience, developing a class of wearable devices for attendees of live music performances that can deliver haptic stimuli and track gestures and/or physiological parameters. These devices are aimed at enriching musical experiences by leveraging the sense of touch and providing new capabilities for creative participation.

Finally, the study by Haynes and colleagues [26] presents “FeelMusic”, a device for the augmentation of music through the haptic translation of some key elements of music. In particular, FeelMusic translates musical features and implements them with “Pump-and-Vibe”, a wearable interface utilizing fluidic actuation and vibration to generate dynamic haptic sensations. The device thus explicitly grounds on the audio-tactile mappings that should enhance auditory experiences. In particular, the squeezer element of Pump-and-Vibe is designed to convey low-pitch and low-frequency elements of music, while the spatially varied motors are capable of conveying faster rhythms and melodies. In this way, the fundamental bass rhythm can be mapped onto the range of pressures available on the squeezer, while the key melody is mapped onto five motors by assigning a note to each motor in order of pitch so that pitch is related to spatial position.

Taken together, these studies show that the translation of auditory features and sensations into the haptic domain is not only possible but also effective in conveying meaningful information to users across different tasks. However, as observed by some of the authors themselves (e.g., ref. [27]), the audio-haptic experience might not be homogeneous across participants, and there might be a lack of appreciation mainly due to the lack of comprehension of the mapping criteria, that is, the relation between what was felt and what was heard. Furthermore, challenges were identified regarding the size and placement of the devices on the body, interferences with concurrent vibrations generated by music signals, limitations on the range of creative controls, and a required training curve.

## 4. Discussion

In this systematic literature review, we examined the integration of wearable technologies in music contexts, categorizing the 23 studies into distinct groups to highlight their main focus areas. The categories we used, i.e., “performance-oriented wearable systems”, “measuring physiological parameters”, “sensory translation/sensory mapping” and “gesture recognition/classification”, were chosen by the authors based on the primary objective of each study; however, it is important to acknowledge that many articles could justifiably fall across different categories due to the interdisciplinary nature of wearable technology applications in music.

The reviewed literature demonstrated the various ways in which wearable systems impact musical contexts, from the design of multi-sensory instruments to systems monitoring key learning parameters. These advancements have opened new avenues for research in music and technology, addressing longstanding challenges within and beyond the field. In education, wearable technologies monitor movements and techniques, offering instant feedback that might accelerate learning. These devices can provide objective, quantifiable data on performance, enabling precise, personalized feedback and targeted improvements over time, allowing also more inclusive musical experiences. Moreover, as shown by studies on sensory mapping, wearable systems can be designed to translate sound into tactile feedback or visual cues, broadening the audience for musical performances and the communication among performers or between performers and audiences.

Additionally, wearables have been often used for creative purposes in artistic performance. For example, wearable sensors can transform a dancer’s movements into musical notes, integrating dance and music into a unified performance, and expanding the boundaries of traditional performance art. However, the review also highlighted potential issues with these technologies. Comfort and ergonomics are major concerns, especially when musicians need to wear these devices for extended periods. Moreover, accurate and reliable data collection is essential to avoid improper technique adjustments, requiring rigorous testing and calibration. Finally, data privacy and security are paramount, as musicians’ performance data must be protected from unauthorized access or misuse.

The different categories of articles considered in this review reveal numerous points of contact and similarities, highlighting the intersections and synergies between different approaches to integrating wearable technologies in the musical context. These overlaps demonstrate how advancements in one area can influence and enhance outcomes in another, leading to a more holistic improvement in musical performance, education, and accessibility. Table 2 provides a summary of these synergies, categorizing the key overlaps between the different technological approaches discussed.

Firstly, many of the wearable technologies examined share the common goal of enhancing musical performance through real-time monitoring and immediate feedback. Wang [10], Li and Wang [11] focused on the use of multi-sensor systems to provide detailed feedback on musicians’ movements. Similarly, Maragliulo et al. [17], Rhodes et al. [18] demonstrate how wearable technology can facilitate new forms of musical interaction. Such a real-time adjustment capability is further enhanced when combined with physiological monitoring, as seen in [16,20], but also in [15], which highlights the interconnection between emotional engagement and physiological responses. This integration ensures that musicians are not only performing at their best technically but are also physically and emotionally resilient.

Inclusivity and accessibility emerge as common themes across several studies. Cavdir and Wang [5], Freire et al. [6] highlight how wearable technologies can democratize music-making, making it accessible to individuals with sensory and motor impairments, thus promoting inclusivity. By utilizing gesture recognition technologies, such as those explored by [17,18], these inclusive instruments can interpret a wide range of physical gestures, making music-making more intuitive and accessible for people with limited mobility or sensory impairments. This research line not only broadens the inclusivity of music education and performance but also leverages advanced technological capabilities to cater to diverse needs.

Finally, the integration of physical movement and musical expression represents a transversal theme. The Serendiptichord by [12] and the integration of wearables in improvisational drumming studied by [13] show how wearable devices can transform the musician’s body into an interactive instrument, expanding expressive and creative possibilities and transforming creative practices. This synergy enhances creativity by enabling new forms of artistic expression that combine movement and music in innovative ways. Furthermore, the real-time feedback mechanisms discussed earlier can be applied here to refine these movements, ensuring that the physical expressions are accurately and effectively translated into the desired musical outcomes.

The intersection between these categories can also foster cross-disciplinary innovation. By bridging together insights and technologies from fields such as biomechanics, computer science, and music education, wearable technologies create opportunities for interdisciplinary research and development. This cross-pollination of ideas can lead to the creation of more sophisticated and versatile wearable devices that are capable of addressing multiple aspects of music performance and education simultaneously. For instance, a project that combines physiological monitoring with gesture recognition and real-time feedback can provide comprehensive tools for both performance enhancement and educational purposes.

Economic and practical accessibility is another area where synergies are evident. While the economic barriers to adopting wearable technologies are significant, the shared advancements across various research efforts can lead to cost-reduction strategies. For example, the widespread adoption of certain technologies in performance settings can drive down costs through economies of scale, making these devices more affordable for educational institutions. Additionally, innovations aimed at simplifying calibration and reducing the need for technical expertise can make these technologies more practical and accessible to a broader range of users. This is particularly important for smaller educational programs and community music initiatives that may lack the resources to invest in expensive equipment and specialized staff.

Finally, these synergies contribute to a more comprehensive approach to music education. By integrating wearable technologies that enhance performance, monitor physiological responses, and foster inclusivity, educators can offer a more holistic and personalized learning experience. Students can benefit from immediate feedback, tailored to their physiological and emotional states, while also having access to inclusive instruments that accommodate diverse needs. This comprehensive approach not only improves learning outcomes but also prepares students for a broader range of performance scenarios, making music education more effective and inclusive. The summarized commonalities presented in Table 2 further underscore the shared advancements across these diverse areas, showcasing the potential for wearable technologies to transform the musical landscape.

To conclude, addressing current challenges related to calibration complexity, technical dependencies, and economic barriers will be crucial for realizing this potential. As we will further discuss in the next sections, particularly in Section 6, future research and development should focus on creating more accessible, user-friendly, and affordable wearable devices, ensuring that their benefits can be widely enjoyed. Through continued innovation and interdisciplinary collaboration, wearable technologies can significantly enhance the intersection of technology and music, making it more interactive, inclusive, and enriching for all participants.

## 5. Limitations of the Reviewed Studies

While the integration of wearable technologies in music contexts has shown significant promise, several challenges and limitations must be addressed to fully realize their potential. The papers reviewed in this study highlight various aspects that require further development and refinement. To better understand the range of wearables discussed in the literature, we created a summary table (Table 3) that captures critical information about the usability, cost, reliability, calibration requirements and need for technical staff. This categorization was not always explicitly detailed in the studies, and in many cases, we inferred these aspects based on the context and descriptions provided by the authors. For instance, usability was assessed based on factors such as the complexity of setup, target users, and the calibration or maintenance demands of each system. Technologies designed for professional use or that require technical expertise were categorized as having lower usability, while those aimed at non-technical users or accessible groups were considered easier to use.

One major limitation is the need for the frequent calibration of wearable devices. Many current wearables, such as EMG systems and multi-node sensors, require precise calibration to function correctly. This process can be complex and cumbersome for non-technical users, posing a barrier to widespread adoption. For example, Maragliulo et al. [17], Rhodes et al. [18] emphasized the importance of accurate gesture recognition, which heavily relies on well-calibrated sensors. Simplifying calibration procedures developing self-calibrating technologies or, even more, integrating the sensors in the instruments themselves instead of the musicians’ body, could significantly enhance usability and accessibility. Furthermore, the effective use of wearable technologies often necessitates the involvement of technical staff. Studies like [10,11] demonstrate the integration of advanced machine learning algorithms and multi-sensor data fusion, which require technical expertise to set up and maintain. Relying on technical support limits the feasibility of these technologies in settings where such expertise is not readily available, such as smaller educational institutions or community music programs. Developing more user-friendly interfaces and robust support systems could mitigate this issue, making these technologies more accessible to a broader audience.

Economic considerations also play a crucial role in the adoption of wearable technologies in music. Although the cost of these technologies has decreased, making them more accessible than before, there are still significant financial barriers, particularly in educational contexts. High-quality wearable devices and the necessary accompanying infrastructure can be prohibitively expensive for many schools and music programs. This economic barrier restricts the ability of these institutions to fully exploit the potential of wearable technologies. Future research and development should focus on cost-reduction strategies, perhaps through the development of more affordable devices or scalable solutions that can be easily implemented in resource-limited settings.

Moreover, issues related to user comfort and the long-term usability of wearable devices remain. Devices that are uncomfortable or intrusive can detract from the performance experience and may not be suitable for extended use. Research by [15,16] highlights the physiological monitoring capabilities of wearables, but these benefits must be balanced with considerations of comfort and practicality. Innovations in materials science and design could lead to more ergonomic and user-friendly devices, enhancing the overall experience for musicians.

Lastly, there is a need for more comprehensive studies that evaluate the long-term impact of wearable technologies on music education and performance. While current research provides valuable insights into their immediate benefits, understanding their sustained impact over time will be crucial for their integration into standard practice. This includes evaluating how these technologies affect learning outcomes, performance quality, and overall musician well-being. For instance, Nijs and Leman [32] conducted a longitudinal study on the use of interactive technologies, specifically the Music Paint Machine, in the instrumental music classroom. Their research highlights how such technologies can significantly enhance learning processes and engagement in music education over extended periods. The findings suggest that interactive and wearable technologies not only improve immediate musical experiences but also contribute positively to long-term educational outcomes, supporting the case for their broader adoption and deeper integration into curricula.

## 6. Future Directions

To address the limitations presented in the above paragraph and fully exploit the potential of wearable technologies in transforming music performance and education, several future directions can be pursued. Simplifying calibration procedures is crucial; research and development should focus on creating self-calibrating technologies or integrating sensors directly into musical instruments. This would eliminate the need for complex calibration procedures, making wearable devices more user-friendly and accessible for non-technical users. Developing more intuitive and robust user interfaces is also essential to reduce dependency on technical staff. Simplified setup and maintenance processes would enable broader adoption in diverse settings, including smaller educational institutions and community music programs. Future research should explore cost-reduction strategies, such as developing more affordable wearable devices or scalable solutions that can be easily implemented in resource-limited settings. This could involve using less expensive materials, optimizing manufacturing processes, or leveraging economies of scale.

Advancements in materials science and design are needed to address issues of comfort and long-term usability. Wearable devices should be made more ergonomic, lightweight, and unobtrusive to ensure they do not detract from the performance experience and can be worn comfortably for extended periods. There is a need for more comprehensive studies that evaluate the long-term impact of wearable technologies on music education and performance. These studies will be crucial in the future and should assess how these technologies affect learning outcomes, performance quality, and overall musician well-being over extended periods. Understanding these long-term effects will be critical for integrating wearable technologies into standard practice.

Encouraging interdisciplinary collaboration between fields such as biomechanics, computer science, and music education can lead to more sophisticated and versatile wearable devices. Cross-disciplinary research can foster innovation and create comprehensive solutions that address multiple aspects of music performance and education simultaneously. Continued focus on developing inclusive technologies that cater to individuals with sensory and motor impairments is essential. This involves creating adaptable and versatile wearable devices that can provide multi-sensory feedback and facilitate participation in music-making for a diverse range of users.

Moreover, future research should emphasize supporting and fostering cross-disciplinary studies that explore ecological contexts for these technologies. This includes designing systems that are increasingly inclusive and not dependent on technical calibration and setup. Such efforts could open the market on a larger scale, including educational and rehabilitative settings that often struggle to implement research developments due to accessibility and cost issues. Expanding the market for wearable technologies to educational and clinical contexts will enhance data collection and allow for the study of real-world usage scenarios. Increased adoption in diverse settings will provide valuable insights into the practical application of these technologies, from performance to education to health related to music contexts. This, in turn, will drive the development of sensors and devices better suited for use in musical contexts, promoting further innovation and refinement.

By addressing the challenges presented in the Section 5 and pursuing these future directions, wearable technologies can become more accessible, practical, and beneficial for a wider range of users. Ultimately, this will enhance the intersection of technology and music, making it more interactive, inclusive, and enriching for all participants.

## 7. Conclusions

This systematic literature review explored the integration of wearable technologies in music contexts, aiming to understand their applications, benefits, and challenges. The review highlighted several key areas where these technologies are making a significant impact.

Wearable sensors and advanced data processing techniques enhance music performance by providing real-time feedback and improving gesture recognition accuracy. These technologies enable musicians to refine their skills through detailed movement analysis, fostering better technical and expressive capabilities. Additionally, innovative applications, such as wearable instruments integrating dance and music, create dynamic and interactive performance environments that merge physicality with musical expression. Beyond enhancing musical performance, wearable technologies have also ventured into the field of movement sonification, a growing field of research that has predominantly evolved within rehabilitation and sports. Movement sonification involves translating physical actions into auditory outputs, providing an innovative method to guide and improve movement patterns through auditory feedback. This approach has been particularly instrumental in rehabilitation settings, where auditory cues help patients correct or enhance specific motor skills. Studies like the one by [33], for example, underscore the diverse applications of real-time movement sonification systems in movement rehabilitation, illustrating its efficacy in supporting patient recovery and skill enhancement across various conditions. Similarly, research by [34,35] delves into sonification use in physiotherapy and sports, highlighting how these sound–movement interactions can significantly aid in both training and rehabilitation processes. They demonstrate that sonification not only facilitates physical therapy by improving movement accuracy and timing but also enhances the engagement and motivation of participants in sports and exercise contexts. While these technologies hold potential for broader applications, their primary development and impact have been most notable in the contexts of rehabilitation and sports performance enhancement, despite the specific musical one.

Wearable technologies are also promoting inclusivity in music-making. Devices designed for the deaf and hard-of-hearing community, for example, as well as those enhancing interactive dance and music performances, demonstrate the potential for these technologies to democratize access to music, making it more inclusive for individuals with sensory and motor impairments. Innovative research by [36] explores ways for the deaf and hard-of-hearing to enjoy music by creating cross-modal music concepts that allow users to explore and customize how they experience music. This approach not only enhances musical enjoyment but also provides a means for deeper engagement with music through alternative sensory pathways. Similarly, Petry et al. [37] have developed music-sensory-substitution systems that support rhythm activities in deaf children, helping them to participate in musical experiences that were previously inaccessible. These systems convert music into formats that can be felt or seen, thus bypassing auditory pathways and tapping into other sensory modalities. Additionally, Jadán et al. [38] discusses the use of sensory substitution to enhance musical perception in the deaf through vibrotactile feedback, where vibrations enable users to ’feel’ the music, thereby enriching their interaction with sound. These studies collectively illustrate how wearable and sensory substitution technologies are being tailored to overcome traditional barriers in music perception and engagement, offering new avenues for inclusive music education and enjoyment.

Relatedly, easy-to-use wearable devices could lead to the design of new musical instruments that are more attractive to a broad group of potential users. These devices allow users to create music in innovative ways, overcoming the barriers or resistance often associated with traditional musical instruments and the learning process.

Research into the physiological aspects of music performance has revealed that wearable sensors can effectively monitor stress and other physiological responses. This capability helps musicians manage performance anxiety and improve overall well-being, providing valuable insights into the physical demands of music performance.

Despite these advancements, several challenges remain. To fully realize the potential of wearable technologies in music, indeed, future research should focus on simplifying calibration processes, developing user-friendly interfaces, and reducing costs through more affordable and scalable solutions. Additionally, innovations in ergonomic design are needed to ensure the long-term comfort and usability of wearable devices. Comprehensive long-term studies are also essential to evaluate the sustained impact of these technologies on music education and performance.

In conclusion, wearable technologies offer transformative potential for music performance and education. By addressing current limitations and continuing to innovate, these technologies can become more accessible, practical, and beneficial, enhancing the intersection of technology and music for a wider range of users. This review provides a foundation for understanding the current landscape and highlights areas for future research and development in this interdisciplinary field.

## Figures and Tables

**Table 1:** List of the 23 papers analyzed in the review. The listed categories are: (A) gesture recognition/classification; (B) measuring physiological parameters; (C) performance-oriented wearable systems; (D) sensory translation/sensory mapping.

N.	Reference		Category
1	Van Der Linden, J.; Schoonderwaldt, E.; Bird, J.; Johnson, R. Musicjacket—combining motion capture and vibrotactile feedback to teach violin bowing. *IEEE Trans. Instrum. Meas.* **2010**, *60*, 104–113.	[20]	A
2	Kusserow, M.; Candia, V.; Amft, O.; Hildebrandt, H.; Folkers, G.; Tröster, G. Monitoring stage fright outside the laboratory: An example in a professional musician using wearable sensors. *Med. Probl. Perform. Artist.* **2012**, *27*, 21–30.	[21]	B
3	Murray-Browne, T.; Mainstone, D.; Bryan-Kinns, N.; Plumbley, M.D. The Serendiptichord: Reflections on the collaborative design process between artist and researcher. *Leonardo* **2013**, *46*, 86–87.	[12]	C
4	Nakra, T.M.; BuSha, B.F. Synchronous sympathy at the symphony: Conductor and audience accord. *Music. Perception Interdiscip. J.* **2014**, *32*, 109–124.	[22]	B
5	van Fenema, E.M.; Gal, P.; van de Griend, M.V.; Jacobs, G.E.; Cohen, A.F. A pilot study evaluating the physiological parameters of performance-induced stress in undergraduate music students. *Digit. Biomarkers* **2018**, *1*, 118–125.	[14]	B
6	Turchet, L.; Barthet, M. Co-design of Musical Haptic Wearables for electronic music performer’s communication. *IEEE Trans. Hum.-Mach. Syst.* **2018**, *49*, 183–193.	[23]	D
7	Maragliulo, S.; Lopes, P.F.A.; Osorio, L.B.; De Almeida, A.T.; Tavakoli, M. Foot gesture recognition through dual channel wearable EMG system. *IEEE Sensors J.* **2019**, *19*, 10187–10197.	[17]	A
8	Dalmazzo, D.; Ramírez, R. Bowing gestures classification in violin performance: a machine learning approach. *Front. Psychol.* **2019**, *10*, 344.	[24]	A
9	Freire, S.; Santos, G.; Armondes, A.; Meneses, E.A.; Wanderley, M.M. Evaluation of inertial sensor data by a comparison with optical motion capture data of guitar strumming gestures. *Sensors* **2020**, *20*, 5722.	[6]	A
10	Rhodes, C.; Allmendinger, R.; Climent, R. New interfaces and approaches to machine learning when classifying gestures within music. *Entropy* **2020**, *22*, 1384.	[18]	A
11	Wang, H. Research on the application of wireless wearable sensing devices in interactive music. *J. Sensors* **2021**, *2021*, 7608867.	[10]	C
12	Provenzale, C.; Di Stefano, N.; Noccaro, A.; Taffoni, F. Assessing the bowing technique in violin beginners using MIMU and optical proximity sensors: A feasibility study. *Sensors* **2021**, *21*, 5817.	[25]	A
13	Haynes, A.; Lawry, J.; Kent, C.; Rossiter, J. FeelMusic: Enriching our emotive experience of music through audio-tactile mappings. *Multimodal Technol. Interact.* **2021**, *5*, 29.	[26]	D
14	Pras, A.; Rodrigues, M.G.; Grupp, V.; Wanderley, M.M. Connecting Free Improvisation Performance and Drumming Gestures Through Digital Wearables. *Front. Psychol.* **2021**, *12*, 576810.	[13]	C
15	Turchet, L.; West, T.; Wanderley, M.M. Touching the audience: musical haptic wearables for augmented and participatory live music performances. *Pers. Ubiquitous Comput.* **2021**, *25*, 749–769.	[27]	D
16	Kim, H.G.; Lee, G.Y.; Kim, M.S. Dual-function integrated emotion-based music classification system using features from physiological signals. *IEEE Trans. Consum. Electron.* **2021**, *67*, 341–349.	[16]	B
17	Sebastiani, L.; Mastorci, F.; Magrini, M.; Paradisi, P.; Pingitore, A. Synchronization between music dynamics and heart rhythm is modulated by the musician’s emotional involvement: A single case study. *Front. Psychol.* **2022**, *13*, 908488.	[15]	B
18	Li, L.; Wang, G. Design and application of interactive music equipment based on wireless wearable sensors. *Sci. Program.* **2022**, *2022*, 4719884.	[11]	C
19	Cavdir, D.; Wang, G. Designing felt experiences with movement-based, wearable musical instruments: From inclusive practices toward participatory design. *Wearable Technol.* **2022**, *3*, e19.	[5]	C
20	Wexler, D.; Yip, J.; Lee, K.P.; Li, X.; Wong, Y.H. A Touch on Musical Innovation: Exploring Wearables and Their Impact on New Interfaces for Musical Expression. *Sensors* **2023**, *24*, 250.	[3]	C
21	Muhammed, Z.; Karunakaran, N.; Bhat, P.P.; Arya, A. Ensemble of Multimodal Deep Learning Models for Violin Bowing Techniques Classification. *J. Adv. Inf. Technol.* **2024**, *15*, 40–48.	[28]	A
22	Li, X.; Shi, Y.; Pan, D. Wearing sensor data integration for promoting the performance skills of music in IoT. *Internet Technol. Lett.* **2024**, *7*, e517.	[29]	A
23	Provenzale, C.; Di Tommaso, F.; Di Stefano, N.; Formica, D.; Taffoni, F. Real-Time Visual Feedback Based on MIMUs Technology Reduces Bowing Errors in Beginner Violin Students. *Sensors* **2024**, *24*, 3961.	[30]	A

**Table 2:** Summary of commonalities across categories of wearable technologies in music contexts. This table highlights key synergies between wearable technologies discussed in Section 4. The similarities are grouped into six categories: real-time monitoring and feedback, inclusivity and accessibility, physical movement and musical expression, cross-disciplinary innovation, economic and practical accessibility, and comprehensive music education. Each category underscores how different studies contribute to the advancement of wearable technologies in music, emphasizing the overlaps in goals, methodologies, and applications across different research areas.

Category	Similarities and Commonalities	Examples
Real-time Monitoring and Feedback	Many wearable technologies aim to provide real-time feedback on musicians’ movements and performance. This helps musicians improve technical skills and manage performance-related challenges.	Studies by [10,11] focus on multi-sensor systems providing movement feedback, while physiological monitoring is highlighted by [15,16].
Inclusivity and Accessibility	Several technologies are designed to make music-making more accessible, particularly for individuals with sensory or motor impairments, using gesture recognition and adaptive instruments.	Cavdir and Wang [5], Freire et al. [6] focus on inclusive instruments for those with disabilities, utilizing gesture recognition technologies from [17,18].
Integration of Movement and Music	Wearable devices that enable musicians to use their body as an instrument, expanding the creative possibilities by translating physical movements into musical expression.	“Serendiptichord” [12,13] demonstrate how wearable devices can transform musicians’ physical movements into interactive instruments.
Cross-disciplinary Innovation	Insights from biomechanics, computer science, and music education are integrated into the development of wearable technologies, fostering interdisciplinary research and new solutions.	Combining physiological monitoring with gesture recognition and real-time feedback is a cross-disciplinary innovation discussed in several studies, e.g., refs. [10,11,15,20].
Economic and Practical Accessibility	Advances in wearable technologies promote cost-reduction strategies and reduce the need for technical expertise, making devices more practical and accessible in educational settings.	Increased adoption could lower costs through economies of scale, simplifying calibration and technical setup. These points are echoed in several research studies, e.g., refs. [5,16,17].
Comprehensive Music Education	The integration of real-time feedback, physiological monitoring, and inclusive technologies provides a more holistic and personalized approach to music education.	Wearable technologies offer tailored feedback that can address both technical and emotional aspects of learning, enhancing educational outcomes for a wider range of students, e.g., refs. [6,13,20].

**Table 3:** Summary of wearable technologies used in the reviewed studies, categorized by key aspects such as usability, cost, reliability, need for calibration, and requirement for technical staff. The information is derived from the detailed analysis of each paper, considering the complexity of setup, target users, calibration needs, and the context of use.

Technology	Usability	Cost	Reliability	Calibration Needed	Technical Staff Needed
Myo armband (e.g., ref. [18])	Easy to use, suitable for gesture recognition in music	Moderate	High	Yes	No
EMG system (e.g., ref. [17])	Moderate complexity, used for foot gesture recognition	High	High	Yes	Yes
Wearable sensors (e.g., [11])	Usable in performance contexts, data integration capabilities	Moderate	High	Yes	Yes
New interfaces for performance (e.g., refs. [12,13])	Moderate complexity, interactive dance-driven music, and music improvisation	Low to Moderate	Moderate	No/Yes	No
Wireless sensing (e.g., ref. [10])	Moderate to high complexity, suitable for interactive music applications	Moderate to High	High	Yes	Yes
Accessible instruments (e.g., ref. [5])	Designed for accessibility, easy to use for DHH community	Low	Moderate	No	No
Physiological monitoring (e.g., refs. [14,15,16,21])	Easy to moderate complexity for stress monitoring	Moderate	Moderate to High	Yes	Yes
Multi-sensor platforms (e.g., ref. [6])	Moderate complexity, used in dance and music performances	Moderate	High	Yes	Yes

## Data Availability

The review protocol can be found here: https://inplasy.com/inplasy-2024-7-0098 (accessed on 2 September 2024). Further inquiries can be directed to the corresponding author.
